# Using photovoice to engage students in a non-major microbiology course

**DOI:** 10.1099/acmi.0.000784.v3

**Published:** 2024-06-26

**Authors:** Bridget G. Kelly

**Affiliations:** 1 Dundalk Institute of Technology, Dundalk, Co. Louth, Ireland

**Keywords:** creativity, microbiology, non-major subject, photovoice, science

## Abstract

In the past decade, it has become increasingly difficult to engage and encourage critical thinking and deeper learning in students who participate in higher education, particularly in non-major subjects. Photovoice is a participatory action research methodology that has been used in community-based research in many different areas including social science, health science and education. In this study, photovoice was used as a pedagogical tool in a third-year BSc Bioscience non-major microbiology module at Dundalk Institute of Technology. In order to ascertain if photovoice was an effective way of engaging these students, a qualitative descriptive methodological approach, in the form of a focus group, was employed. Six of the 13 students who took the module participated in the focus group, reporting a positive experience overall of using photovoice. Further analysis of the focus group data resulted in the overarching theme of choice, with creativity and critical thinking and research skills as sub-themes to emerge. These findings suggest that photovoice is an effective way to engage students in microbiology as a non-major subject. However, as it was a small sample size, future research would need to use a larger cohort of students to provide further evidence of using photovoice as a pedagogical engagement tool for non-major subjects.

## Data Summary

No new data, tools or software has been generated or is required for this work to be reproduced.

## Introduction

Teaching non-major subjects, i.e. subjects that only form a minor part of a degree, at Higher Education Institutions (HEIs) has been found to be difficult in several instances, and many approaches have been used in order to overcome this. Challenges include the lack of interest students have in the subject, students not liking the subject as well as students not seeing the relevance of the subject in question. All of these issues can lead to a general disengagement in the non-major subject. This has been found on many occasions, including a study by Burks [1], where students who undertook a non-major introductory biology course at Bowie University in Maryland, USA, lacked connection with the course and viewed it as irrelevant. Here, a project-based community approach was used to address the issue, resulting with the students engaging with the material of the non-major introductory biology course and improving their overall results in that course. Another example is in a study by Federici [2], where non-computer science majors, who had a computer science component as part of their health science qualification at the University of Cagliari, Italy, did not see the significance of computer science to their degree. Federici found that in this case study, by allowing students to be involved in customizing the computer science course during the introductory lectures, the course was deemed relevant and important for their future careers as health professionals.

Many researchers have devised other ways to address these issues. A prime example is in mathematics, which has a role in most scientific disciplines and is often cited as a difficult topic for students to understand and so students tend to lose interest. Marchisio *et al*. [3] changed the content of their first-year mathematics course to include relevant applications and problem solving, allowing for active and collaborative learning to take place amongst students. Similar issues have arisen with statistics, a subject that is increasingly important not just in academic circles, but increasingly in wider society. Bromage *et al*. [4] examined the issues surrounding the teaching of statistics, and reviewed current ideas to address this such as using interactive learning resources, computational tools and digital resources. To address similar concerns in other disciplines, comics were used in a number of ways to make non-major subjects more interactive and interesting, for example in biology [5, 6]. Comics have also been used in teaching science to students in Turkey [7] and to communicate microbiological topics to the general public [8]. Another approach was used by Mullen *et al*. [9], where they incorporated the writing of a children’s book learning activity into non-major chemistry courses which helped students think about chemistry in a different way. All of these methods encompass the active learning process where students are at the centre and are directly engaged in their learning; these methods also promote critical thinking, deeper learning and understanding of the particular topic [10,11]. Active learning has also been found, in a recent literature review, to positively impact student wellbeing as well as nurturing transferable skills for their future careers [12].

Microbiology is taught at many HEIs across Europe and the world. In Ireland, many institutions offer microbiology as a major degree course, and as an important element of other major awards. At Dundalk Institute of Technology, microbiology is offered as a compulsory non-major subject across the science, agriculture, nursing and veterinary nursing disciplines. These students have one or two microbiology modules across these degree courses and have many other modules apart from microbiology. Issues mentioned previously such as lack of engagement, lack of interest as well as an absence of critical analysis in microbiology have all been informally noted across the various student cohorts participating in these degrees, but there is a lack of studies in the literature that tackle this. In one older study, Fass [13] successfully addressed issues around engagement and scientific literacy with microbiology as a non-major subject at Beloit College, Wisconsin, USA, by using active learning methodology around the topic of emerging diseases which students were naturally curious about. In more recent study, Bull *et al*. [14] addressed the student engagement issue in a non-major introductory large microbiology class by introducing an innovative intervention to promote active learning where students were tasked with creating virtual posters around different themes.

As mentioned above, students who study microbiology as a non-major subject at Dundalk Institute of Technology have a variety of other modules to complete for their degrees; their attention and focus is divided in many different directions; and they have a variety of assignments and different teaching and learning styles from many lecturers. With all of this, it can be difficult to get students enthused and engaged about some modules, including microbiology, and from this non-engagement, students also seem to have a difficulty with critical thinking skills. These concerns have been informally noted at staff programme board meetings. To address these issues, the current study looked at using photovoice as an innovative way to engage students in microbiology as a non-major subject at Dundalk Institute of Technology.

Photovoice has been used as a participatory action research method since 1994, when Wang and Burris [15] first described this technique. Participatory action research is a research method which is concerned with ensuring that knowledge development is accessible to all with the involvement of community in the whole research process [16]. In photovoice, participants are invited to use photography to detail part of their lives through photographs and produce a narrative around the images created [17,18]. Originally called ‘photo novella’, photovoice was first used to document the struggles that women from Yunnan Province in China experience as part of their daily lives [15]. The use of photovoice specifically as a pedagogical tool allows students to document issues relating to an academic topic from their point of view. It gives students novel and interesting ways to see the world around them. It has been used in different educational settings and with different subject areas ranging from environmental science, chemistry, design education to health science. Photovoice has been used in a highschool setting, as both a pedagogical tool and an action research method by new teachers to find out prior knowledge of science outside the school setting in 14 and 15 year olds [19]. By using reflection and whole class discussion based on the images produced by students, the teachers were able to develop novel teaching skills which more encompassed the disparate student interest in science within the class.

Photovoice has also been used to elicit more interest in science with students who are not studying science as a major part of their degrees. Cook and Quigley [20] used photovoice with non-major science students as a way of connecting with their community and to aid understanding of the topic. In their study, students who were training to be elementary teachers took photos which represented an environmental issue and discussed the photos with both their classmates and local community members. From this, the student teachers became more empowered and engaged with local issues, and they connected to the science in their place. The resulting photos were assessed on criteria which included: the quality of the photo; and relevance of the photo to the topic and level of discussion around the topic. Photovoice was used by Waters and Cook [21] to engage non-science majors with environmental science in a private Midwestern US University. Part of the student academic assignment was to take photos in their communities with a view to identifying important environmental issues, as well as producing an annotated bibliography which deepened their understanding and awareness of solutions relating to the issues that were raised. Another aspect of this project was the discussion of the chosen photos with community members at an external community event. Using photovoice in this way for the environmental science curriculum allowed students to become more confident in their knowledge of environmental science and to be directly involved in their learning process, switching the focus of assignments from learning by rote to deepening content knowledge and reflection. With undergraduates who were studying chemistry as a non-major subject, Stroud [22] found that photovoice assignments were an effective constructivist student-centred activity and a change from the posivisitic teacher-centred type of lecture and practical that is the norm. When the students completed their photovoice projects, they could see the relevance of chemistry in the world around them making them more enthusiastic and engaged in the topic.

Photography and visuals have been used to encourage students to think more critically about, and to engage more with, a topic in a university setting, like in the study by Walter and Baller [23], who found that using photography assignments such as photovoice with undergraduate students from two health science courses allowed the students to increase their level of engagement and critical reflection. In the study, students took different photos over a 4 week period and selected ones that represented the environmental impact of alcohol and tobacco use around campus. A review by Haultain [24], which examined how photographs were used in a number of studies at different HEIs, found that there are many benefits to using a variety of photographic methods (including photovoice) in teaching and learning: enhancement of students’ learning and engagement; empowering and motivating students; increasing student creativity and enjoyment; encouraging critical thinking and collaborative learning; and consolidation of learning and as a means to bridge any gaps in the lecture material. In studies that have specifically used photovoice as a teaching and learning tool, where students are asked to take photographs of a particular topic and how it resonates with them, similar advantages have been found [17].

As microbiology is a non-major subject as part of many degree courses at Dundalk Institute of Technology and students have a plethora of other subjects to contend with, novel pedagogical tools are needed to sustain interest in the topic. Photovoice may be a solution to aid not just engagement in the topic, but also to promote deeper learning and critical thinking, as found in previous cases. This study used photovoice as a pedagogical tool in a third-year microbiology module, and aimed to address the research question:

- Is photovoice an effective way to engage non-major students in microbiology?

In order to answer this question, the student experience of using photovoice was assessed using a focus group and the data were analysed to assess the effectiveness of using this technique to enhance deeper learning and critical thinking. Conclusions drawn from this study may be useful in the design of non-major microbiology courses in a variety of different fields.

## Methods

### Methodology

This study investigated whether photovoice is an effective way to engage students who took microbiology as part of their degree at Dundalk Institute of Technology. As experiences differ from student to student, a qualitative descriptive methodological approach was employed. A focus group was used because researchers can glean a broad range of information, as well as rich insights from focus groups, besides the facts that could be gathered from just using a survey [25,26]. Focus groups have also been utilized in a similar study, where photovoice was used as a pedagogical tool to enhance critical thinking in health science students, which yielded considerable insight into the student experience of using photovoice as a pedagogical tool [27].

### Participants

Participants were third-year students of BSc Bioscience at Dundalk Institute of Technology, who take one module of microbiology amongst a variety of other science modules such as biotechnology, immunology, analytical science and good manufacturing practice. The 13 students that took the microbiology module, taught in the first semester of 2022–2023, were invited to participate in a focus group in the second semester of 2022–2023, once the end of semester exam for the module was completed, confirmed and the results were processed. Two of the students were unable to participate in the focus group as they were Erasmus students only present for one semester and they returned to their countries at the end of the first semester. Six students participated in the focus group, which was facilitated by a lecturer from another department who does not teach the students, to keep participant anonymity. Participants were given a participant information leaflet advising of the purpose of the focus group, the benefits/risks, location, date and time, as well as outlining the right to opt out of the study at any time.

### Task

As part of the microbiology module, students were given a photovoice assignment worth 20 % of the overall module grade, where they were asked to take two photographs which represent ‘microbiology in our world’ and to write a narrative paragraph (350–400 words) explaining why they took the photos. The narrative paragraph was guided by questions linked to the letters in PHOTO (Table 1), as described by Haffejee [27].

**Table 1.** Questions associated with the letters in PHOTO

Questions associated with the letters in PHOTO
Describe the **P**icture
What is **H**appening in the picture and how does it relate to microbiology in our world?
Why did you take a picture **O**f this?
What can the picture **T**ell us about the importance of microbiology?
How does this picture provide **O**pportinities for discussion regarding microbiology in society?

Students were given the assignment brief within the first 2 weeks of semester 1 of 2022–2023 and the assignment was submitted later in the semester. The photovoice assignment was graded according to a rubric designed for this study adapted from previous studies [21,22, 27].

### Procedure

The focus group interview, held in February 2023, was recorded using a hand-held recording device and transcribed manually by the author. The duration of the focus group was 16.42 min, which transcribed to approx. 3 000 words in total. During the course of the focus group, the moderator had to be more vocal and ask repeating probing questions as the participants were a quiet group and quite reticent to speak.

The focus group questions that were given to the moderator are outlined in Table 2.

**Table 2.** Focus group questions

Questions for the focus group
General perceptions and experiences of using photovoice
Was it easy/difficult to obtain the photos and decide what to use?
Were there any barriers that you faced to obtaining the photos?
How did you feel about this assignment?
Were there any benefits to gathering this information yourself rather than being given the information?
What did you learn from this assignment?

## Results

### Data analysis

The six steps outlined in the table below (Table 3), adapted from Braun and Clarke [28], were used to analyse the data, with guidance from the worked examples by Maguire and Delahunt [29] and Byrne [30].

**Table 3.** Description of steps used to analyse data in this study

Steps to analyse data	Steps used in this study
1. Become familiar with the data	Researcher first listened to the focus group recording once before verbatim transcription of the recording. Transcript was re-read numerous times, with initial thoughts noted
2. Generate initial codes	Researcher started to organize the data in a meaningful way, making initial codes
3. Search for themes	When relevant data items were coded, researcher searched for themes to emerge
4. Review themes	Researcher reviewed themes to ensure that all coded items were represented within these themes
5. Define themes	Researcher refined themes in relation to the research question
6. Write up the report	Researcher described the themes in the final article

Analysis of the data was driven by a top-down theoretical thematic analysis approach [28] guided by the overarching research question: ‘Is photovoice an effective way to engage students in microbiology?’. Because of this, open coding was used and codes were developed and modified as the coding process was worked through.

A number of themes were initially identified from the focus group data. Following reflection and review on these initial themes over a period of time an overarching theme of choice was identified within the transcript, consisting of two sub-themes: (1) Creativity and (2) Research and critical thinking skills.

### Choice as overarching theme

Choice as an overarching theme emerged from the data. The idea of student choice in this study allows the two sub-themes of creativity and critical thinking to occur. This aligns well with previous studies, where it has been found that if students are given some level of choice in their assessment, they take responsibility for their own learning, which positively impacts their engagement and motivation for the task, the cultivation of critical thinking skills as well as creativity. Pretorius *et al*. [31] found that critical and higher order thinking skills were positively impacted when students were given a choice on assessment and this was also discussed in the report on the provision of choice by Biernacki [32]. Lasley [33], in her piece around course design, commented that by giving students a choice in assignment, creativity and motivation increased with her own students. These data and the emerging theme of choice and sub-themes of creativity and critical thinking suggest that photovoice is an effective way to engage non-major students in microbiology.

### Creativity

Students of microbiology traditionally have many laboratory reports and other types of written reports to complete as assessments for their modules. These reports usually have to be written on a particular topic in a standardized scientific way. Creativity emerged as one of the sub-themes of this particular study; students found using photovoice as an assignment strategy was a ‘different way of learning’ and because of this ‘you had to be creative’ [Participant 1], and another student commented that it was a ‘nice break from lab reports’ [Participant 3]. One student thought that getting a ‘chance to go … beyond the lecture notes’ allowed them to ‘creatively, critically think of what we’re interested in in terms of microbiology, even though it’s not like, covered in the lectures’ [Participant 4]. Students got to decide what to photograph and had an opportunity to think outside the box, with one student commenting that ‘you had nearly nothing to follow, so I found it quite creative, in a way’ [Participant 2]. Uncertainty, which initially was considered a sub-theme, fits in under the banner of creativity, as in order to be creative, you need to do something different and ‘there is no creativity without uncertainty’ as Beghetto [34] asserted in a keynote address. As mentioned before, assignments such as lab reports are based on particular topics and written in a defined manner. In the photovoice assignment, students were asked to photograph something that represented microbiology to them; students were not used to doing this and this brought a level of uncertainty and discomfort. A few students felt that they found the photovoice assignment to be scary ‘because we’ve never done anything like this before’ and ‘I was just unsure at the start like, how to approach it, cos we hadn’t done anything like that before, so I was a wee bit anxious’ [Participant 1]. By using photovoice as an assignment for microbiology, the traditional mode of assessment for the module was disrupted, leading to uncertainty which in turn lead to creativity, similar to how Walter *et al*. [23] used photovoice in their study with healthcare students. In the current study, this spark of creativity enabled students to be more engaged and interested in microbiology.

### Critical thinking and research skills

In order to complete the photovoice assignment, students had to engage in critical thinking and research skills. Students remarked that they had to think ‘beyond the lecture notes’ [Participant 4], ‘to critically think of what we’re interested in’ as well as ‘think about something related to microbiology before you can just take a picture’ [Participant 6] to finish their assignment. As this assignment was not just about taking a photograph of something and students were required to write a paragraph about why the photograph was relevant to microbiology, students had to utilize research skills and ‘look into things that I was interested in and find out more about it’ [Participant 1] and to ‘think why you want to take the photo’ [Participant 5]. The research skills gained meant that students ‘got some experience in terms of researching ourselves, and we used that in other modules as well’ [Participant 3]. Students found that this assignment allowed them to consider a wider view of microbiology, how it can ‘relate to the real world’ [Participant 5], and how ‘it’s one thing to learn about different topics in the class, but having to think about it and like, its impact on you or other people in real life was really interesting’ [Participant 2]. Students commented that using photovoice ‘made me look into things that I was interested and find out more about it’ [Participant 1]. Another student used the assignment ‘to think about how it was impacting me’ [Participant 2], in relation to microbiology. One important aspect to come out of the focus group is that students realize that ‘microbiology is very valuable to our lives’ [Participant 5]. Having to write a narrative piece with a strict word count about the photo that students had taken assisted the development of writing skills, and allowed students to ‘focus on what was really important’ [Participant 2]. Students took ownership of this assignment as it ‘promoted, like, more of, like, self-learning’ [Participant 1]. By doing the photovoice assignment, students were empowered be more engaged in their learning and this helped to develop critical thinking and research skills.

### Deeper learning

Deeper learning did not arise as a major theme during the discussions in the focus group, but, at the end of the session, the moderator asked the participants if they thought they had ‘retained the information quite well, and all of the learning that you got from it?’, with most of the participants agreeing. This suggests that perhaps deeper learning may have taken place, which could be attributed to the creativeness of the assignment, as previous studies have shown that creativity encourages critical reflection, adaptability and deeper learning, all integral skills for a healthy and prosperous society [35].

### Overall experience of photovoice

At the end of the focus group, the moderator asked the participants if there were any other comments that they would like to add regarding their experience with photovoice as an assignment. Everyone agreed that is was a positive experience, with some students commenting that ‘it was great’ [Participant 5] and that the ‘overall experience I think was good’ [Participant 1].

## Discussion

The aim of this study was to assess whether photovoice is an effective way to engage students in microbiology as a non-major subject at Dundalk Institute of Technology. This was achieved by assessing the student experience of using photovoice as a pedagogical tool in the form of a focus group. Students agreed that it was a positive experience and the overarching theme of choice emerged with creativity, and critical thinking and research skills emerging as sub-themes.

For the photovoice assignment, students were given the choice to photograph something that represented their view of ‘microbiology in our world’ and this choice gave students the opportunity to be creative, and to use critical thinking and research skills in their decisions around what to photograph. By having this choice in what to photograph, students became actively engaged in the process and became responsible for their own learning. This is in line with previous studies by Pretorious *et al*. [31], who found that by giving students some level of choice in their assessment, they will take responsibility for their own learning which positively impacts their engagement in the task. In this current study, the choice given to students as part of the photovoice assignment enabled their learning to transition from passive learning to active learning. Thus, photovoice was found to be an effective active learning strategy as students became directly engaged in the task allowing for critical thinking to occur, similar to the studies on active learning strategies by Gleason *et al*. [10] with pharmacy students and Hernández-de-Menéndez *et al*. [11] with engineering students.

Photovoice was also shown to be an effective strategy to engage learners in microbiology as a non-major subject, similar to what was found with chemistry as a non-major subject in the study by Stroud [22] and with environmental science as a non-major subject by Waters and Cook [21]. However, in both of these studies, evaluation of engagement was not investigated directly from the student experience, but by the photos produced on the topics in question and the discussions arising from these photographs, so is not clear if choice, critical thinking and creativity were important elements in engagement in those cases.

Deeper learning has also been reported as a consequence of active learning [10,11]. When students were directly asked by the moderator at the end of the study if they retained the information that they had learnt from photovoice many months after the assignment, they agreed they had. This suggests that a deeper learning may have taken place, but since this did not come up naturally with the participants during the course of the focus group, and there was no method employed to measure it, it is unclear if actual deeper learning occurred in this instance. Further research into the use of photovoice as a pedagogical tool is needed to assess any impact on deeper learning.

There were a number of limitations that should be noted. This study represented one module of microbiology in the third year of the BSc Bioscience course at Dundalk Institute of Technology, so data obtained could not be considered representative of the other degree courses for which microbiology is a non-major subject at the institute. Students self-selected to participate in the focus group, so perhaps only those students who had a good experience were likely to participate in the focus group. Another limitation is that data from the focus group cannot be more broadly applied due to the small selected sample size. The photovoice task was a different type of assignment than the standard lab report or essay that science students would normally be given, and it had a novelty value to it, which may explain why students felt positively about it. If the photovoice task was repeated over different stages of the degree courses, students may think differently. However, the data from the focus group do present some insight into how the students experienced photovoice as a pedagogical tool.

Ideally, future studies into using photovoice as a pedagogical tool for non-majors in microbiology would use a larger cohort of students as well cohorts of other students within Dundalk Institute of Technology who have microbiology as part of their degree courses, for example agriculture students. This could provide further evidence of using photovoice as a pedagogical engagement tool for non-major subjects.

## Conclusion

The issue of engagement in non-major subjects in many degree courses is a problem. This study showed that photovoice is an effective way to engage students in microbiology as a non-major subject of the BSc Bioscience degree at Dundalk Institute of Technology. The use of photovoice as an assignment allowed students choice, which encouraged creativity and critical thinking and research skills to be developed.

## Abbreviation

HEI: Higher Education Institute
